# 2-(Aminocarbonyl)hydrazin-1-ium 6-carboxy­picolinate

**DOI:** 10.1107/S1600536809020601

**Published:** 2009-06-06

**Authors:** Mohammad Idrees, Shah Mohammad Shadab, Sarvendra Kumar

**Affiliations:** a Department of Chemical Engineering, Aligarh Muslim University, Aligarh, India; b Indian Institute of Technology, Kanpur, India

## Abstract

In the crystal structure of the title proton-transfer compound, CH_6_N_3_O^+^·C_7_H_4_NO_4_^−^, O—H⋯O and N—H⋯O hydrogen bonds are formed respectively between the cations and the anions, each component affording a supra­molecular chain along the *c* axis. The cation and anion chains are further linked by N—H⋯O and N—H⋯N hydrogen bonds. A π–π inter­action is also observed between the pyridine rings; the inter­planar separation and the centroid–centroid distance are 3.3425 (6) and 4.6256 (11) Å, respectively.

## Related literature

For general background, see: Desiraju (1997 ▶); Braga *et al.* (1998 ▶). For related structures of proton-transfer compounds, see: Moghimi *et al.* (2002 ▶, 2003 ▶, 2007 ▶); Aghabozorg *et al.* (2008 ▶); Soleimannejad *et al.* (2008 ▶).
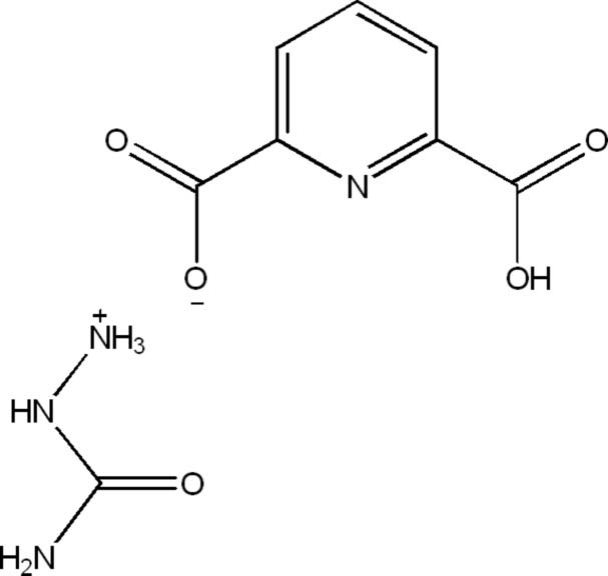


## Experimental

### 

#### Crystal data

CH_6_N_3_O^+^·C_7_H_4_NO_4_^−^
*M* *_r_* = 242.20
Monoclinic, 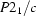

*a* = 7.9553 (11) Å
*b* = 14.965 (2) Å
*c* = 8.5510 (11) Å
β = 97.305 (2)°
*V* = 1009.7 (2) Å^3^
*Z* = 4
Mo *K*α radiation
μ = 0.13 mm^−1^
*T* = 293 K
0.30 × 0.28 × 0.25 mm

#### Data collection

Bruker SMART CCD diffractometer
Absorption correction: multi-scan (**SADABS**; Sheldrick, 2004 ▶) *T*_min_ = 0.961, *T*_max_ = 0.967
10168 measured reflections
1980 independent reflections
1865 reflections with *I* > 2σ(*I*)
*R*_int_ = 0.023

#### Refinement

*R*[*F* ^2^ > 2σ(*F* ^2^)] = 0.041
*wR*(*F* ^2^) = 0.102
*S* = 0.85
1980 reflections
170 parameters
H atoms treated by a mixture of independent and constrained refinement
Δρ_max_ = 0.20 e Å^−3^
Δρ_min_ = −0.25 e Å^−3^

### 

Data collection: *SMART* (Bruker, 2001 ▶); cell refinement: *SAINT* (Bruker, 2001 ▶); data reduction: *SAINT*; program(s) used to solve structure: *SHELXS97* (Sheldrick, 2008 ▶); program(s) used to refine structure: *SHELXL97* (Sheldrick, 2008 ▶); molecular graphics: *SHELXTL* (Sheldrick, 2008 ▶); software used to prepare material for publication: *SHELXTL*.

## Supplementary Material

Crystal structure: contains datablocks I, Global. DOI: 10.1107/S1600536809020601/is2423sup1.cif

Structure factors: contains datablocks I. DOI: 10.1107/S1600536809020601/is2423Isup2.hkl

Additional supplementary materials:  crystallographic information; 3D view; checkCIF report

## Figures and Tables

**Table 1:** Hydrogen-bond geometry (Å, °)

*D*—H⋯*A*	*D*—H	H⋯*A*	*D*⋯*A*	*D*—H⋯*A*
N2—H01*A*⋯O1^i^	0.89	2.00	2.7551 (17)	141
N2—H01*A*⋯N1^i^	0.89	2.21	2.9361 (17)	139
N2—H01*B*⋯O4^ii^	0.89	2.04	2.8781 (19)	156
N2—H01*C*⋯O1	0.89	1.84	2.7258 (18)	176
N4—H04⋯O2^iii^	0.83 (2)	2.22 (2)	2.993 (2)	155 (2)
N3—H08⋯O5^i^	0.88 (2)	2.06 (2)	2.8362 (19)	146.2 (19)
N3—H08⋯O1^i^	0.88 (2)	2.49 (2)	2.9319 (18)	112.0 (16)
O3—H09⋯O2^iv^	0.84 (2)	1.79 (2)	2.6109 (16)	165 (2)
N4—H06⋯O5^i^	0.88 (2)	2.05 (2)	2.869 (2)	153.5 (19)

Symmetry codes: (i) 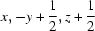; (ii) 
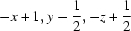
; (iii) 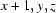; (iv) 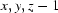.

**Fig. 1.**
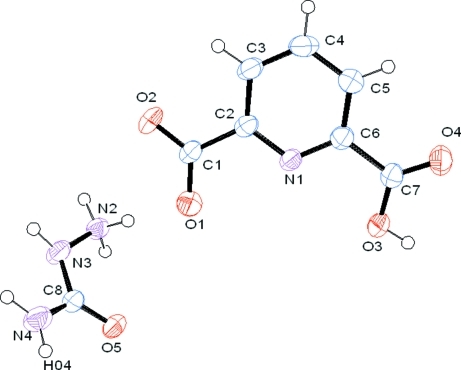
ORTEP diagram of the title compound, showing displacement ellipsoids at the 50% probability level for non-hydrogen atoms.

**Fig. 2.**
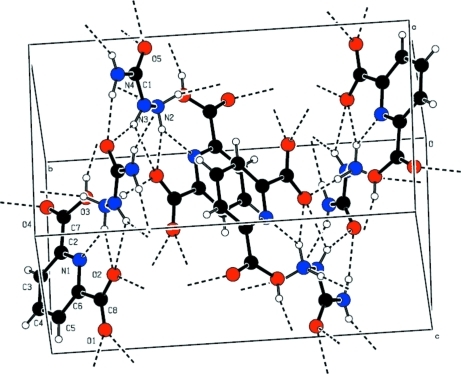
Packing diagram, showing molecules linked by N—H···O, O—H···O and N—H···N hydrogen bonds (dashed lines).

**Fig. 3.**
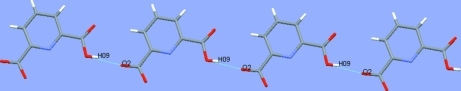
Supramolecular cation chain formed by O—H···O hydrogen bonds (dashed lines).

**Fig. 4.**
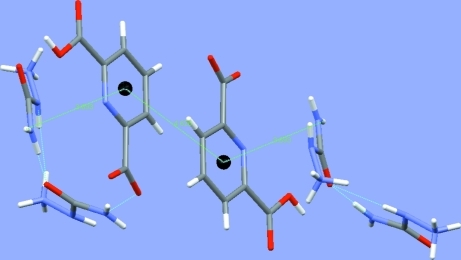
π–π interaction between the benzene rings.

## supplementary crystallographic information

### Comment

In the synthesis of crystal structure by design, the assembly of molecule unit in predefined arrangement is a key goal (Desiraju, 1997; Braga *et al.* 1998). The water soluble, proton transfer compounds can function as suitable ligands in the synthesis of metal complexes. In general, molecular association between carboxylic acid and a Lewis base results in more hydrogen bonding association with considerable stability upon a structure making process. This is because functionalized carboxylic acids and amines can enhance the intermolecular forces between the obtained cationic and anionic fragments, and these interactions can provide a large part of the stabilization energy of resulting self assembly systems (Moghimi *et al.*, 2003, 2007). Proton transfer from carboxylic acid to different amines has been reported (Moghimi *et al.* 2002; Aghabozorg *et al.*, 2008; Soleimannejad *et al.*, 2008). Herein, we report a novel PTC that have been synthesized using pyridine-2,6-dicarboxylic acid and hydrazinecarboxamide at room temperature and its crystal structure.

In the crystal structure of the compound, intermolecular hydrogen bonds link the molecules to form a proton transfer supramolecular framework. These hydrogen bonds help in the stabilization of the resulting supramolecular structure of the compound (Table 1). The molecular structure of the title compound is shown in (Fig. 1). The crystal structure shows that a single proton from each of the carboxyl groups was transferred to the hydrazinecarboxamide. Thus, the negative charges of monoanionic pyridine-2,6-dicarboxylate groups, (pyH)-, are neutralized by a mono protonated hydraziniumcarboxamide fragment. The C–O distances for this compound support the existence of both ionic (COO) and non-ionic COOH group in the crystal structure of a new proton transfer system. The distance of ionic C—O in carboxylate ion is in the range of 1.250 Å due to resonance but in carboxylic acid group of pyridine-2,6-dicarboxylic acid has a deviation of 0.1 Å. The distance of C—O in carboxlate and carboxylic acid are quite similar to the report (Aghabozorg *et al.*, 2008). The relatively short bond distances of C1–O2 [1.250 (18) Å] and C7–O7 [1.211 (19) Å] confirm the presence of double bonds. In the crystal structure of title compound, a number of N–H—O, N–H—N and O–H—O hydrogen bonds, with D—A ranging from 2.612 (2) to 2.994 (3) Å are observed (Fig. 2). The carboxylate group of one pyridine-2,6-dicarboxylic acid are bonded to OH of another pyridine-2,6-dicarboxylic acid through hydrogen bonding (Fig. 3) with distance of 1.791 Å. In the packing diagram, crystal structure shows hydrogen bonding, ion pairing, π-π stacking and van der Waals interactions. The π–π stacking interactions exist between the two pyridine rings and between the pyridine ring and the NH_2_ group of the cation with a centroid-centroid distance of 4.626 Å and a π–HN distance of 3.693 Å, respectively (Fig. 4). These interactions result in the formation of a supramolecular structure.

### Experimental

Pyridine-2,6-dicarboxylic acid was purchased from Merck and used as received. Solvents dimethyl formamide (LobaChemie) and methanol (Qualigens) were used as received. Pyridine-2,6-dicarboxylic acid (11.69 g, 70 mmol) dissolved in methanol-water (100:175 ml) in hot condition over a period of 1 h 30 min. Semicarbazide hydrochloride (7.81 g, 70 mmol) dissolved in DMF-methanol (150:100 ml) was added to the pyridine-2,6-dicarboxylic acid solution in portions with continuous stirring. The reaction mixture was allowed to cool at RT with continuous stirring. The reaction mixture was stirred for 48 h. However, no precipitation was seen.  Subsequently, it was allowed to stand for 24 h. Transparent crystalline compound was seen at the bottom of the flask, which was separated by decanting the solution. Crystals were washed with methanol and dried in desiccator. The crystals are stable at room temperature.

### Refinement

H atoms attached to atoms N3, N4 and O3 were refined freely. Other H atoms were introduced in calculated positions (C—H = 0.93 and N—H = 0.89 Å) and treated as riding, with *U*_iso_(H) = 1.2*U*_eq_(C) or 1.5*U*_eq_(N).

### Crystal data

**Table:**

CH_6_N_3_O^+^·C_7_H_4_NO_4_^−^	*F*(000) = 504
*M**_r_* = 242.20	*D*_x_ = 1.593 Mg m^−^^3^
Monoclinic, *P*2_1_/*c*	Melting point: 475 K
Hall symbol: -P 2ybc	Mo *K*α radiation, λ = 0.71073 Å
*a* = 7.9553 (11) Å	Cell parameters from 7532 reflections
*b* = 14.965 (2) Å	θ = 2.6–26.0°
*c* = 8.5510 (11) Å	µ = 0.13 mm^−^^1^
β = 97.305 (2)°	*T* = 293 K
*V* = 1009.7 (2) Å^3^	Block, colorless
*Z* = 4	0.30 × 0.28 × 0.25 mm

### Data collection

**Table:**

Bruker SMART CCD diffractometer	1980 independent reflections
Radiation source: fine-focus sealed tube	1865 reflections with *I* > 2σ(*I*)
graphite	*R*_int_ = 0.023
phi and ω scans	θ_max_ = 26.0°, θ_min_ = 2.6°
Absorption correction: multi-scan (*SADABS*; Sheldrick, 2004)	*h* = −9→9
*T*_min_ = 0.961, *T*_max_ = 0.967	*k* = −18→18
10168 measured reflections	*l* = −10→10

### Refinement

**Table:**

Refinement on *F*^2^	Primary atom site location: structure-invariant direct methods
Least-squares matrix: full	Secondary atom site location: difference Fourier map
*R*[*F*^2^ > 2σ(*F*^2^)] = 0.041	Hydrogen site location: inferred from neighbouring sites
*wR*(*F*^2^) = 0.102	H atoms treated by a mixture of independent and constrained refinement
*S* = 0.85	*w* = 1/[σ^2^(*F*_o_^2^) + (0.0606*P*)^2^ + 0.7523*P*] where *P* = (*F*_o_^2^ + 2*F*_c_^2^)/3
1980 reflections	(Δ/σ)_max_ < 0.001
170 parameters	Δρ_max_ = 0.20 e Å^−^^3^
0 restraints	Δρ_min_ = −0.25 e Å^−^^3^

### Special details

**Table:**

Geometry. All e.s.d.'s (except the e.s.d. in the dihedral angle between two l.s. planes) are estimated using the full covariance matrix. The cell e.s.d.'s are taken into account individually in the estimation of e.s.d.'s in distances, angles and torsion angles; correlations between e.s.d.'s in cell parameters are only used when they are defined by crystal symmetry. An approximate (isotropic) treatment of cell e.s.d.'s is used for estimating e.s.d.'s involving l.s. planes.
Refinement. Refinement of *F*^2^ against ALL reflections. The weighted *R*-factor *wR* and goodness of fit *S* are based on *F*^2^, conventional *R*-factors *R* are based on *F*, with *F* set to zero for negative *F*^2^. The threshold expression of *F*^2^ > σ(*F*^2^) is used only for calculating *R*-factors(gt) *etc*. and is not relevant to the choice of reflections for refinement. *R*-factors based on *F*^2^ are statistically about twice as large as those based on *F*, and *R*- factors based on ALL data will be even larger.

### Fractional atomic coordinates and isotropic or equivalent isotropic displacement parameters (Å^2^)

**Table:**

	*x*	*y*	*z*	*U*_iso_*/*U*_eq_	
C1	0.47854 (19)	0.35088 (10)	0.52831 (17)	0.0322 (3)	
C2	0.37085 (18)	0.41208 (10)	0.41520 (17)	0.0312 (3)	
C3	0.2536 (2)	0.47012 (12)	0.46794 (19)	0.0383 (4)	
H3	0.2400	0.4728	0.5743	0.046*	
C4	0.1580 (2)	0.52359 (13)	0.3594 (2)	0.0444 (4)	
H4	0.0805	0.5639	0.3919	0.053*	
C5	0.1785 (2)	0.51663 (12)	0.2019 (2)	0.0400 (4)	
H5	0.1142	0.5512	0.1262	0.048*	
C6	0.29775 (18)	0.45658 (10)	0.15969 (17)	0.0316 (3)	
C7	0.31929 (19)	0.44623 (10)	−0.01161 (18)	0.0325 (3)	
C8	0.99077 (19)	0.22252 (10)	0.68173 (17)	0.0330 (3)	
N1	0.39422 (15)	0.40574 (8)	0.26364 (14)	0.0302 (3)	
N2	0.70619 (16)	0.17789 (9)	0.68390 (15)	0.0335 (3)	
H01A	0.6308	0.1692	0.7509	0.050*	
H01B	0.7228	0.1269	0.6346	0.050*	
H01C	0.6675	0.2192	0.6134	0.050*	
N3	0.86050 (17)	0.20697 (11)	0.76760 (17)	0.0426 (4)	
N4	1.1225 (2)	0.26456 (12)	0.75923 (19)	0.0475 (4)	
O1	0.59699 (15)	0.31012 (8)	0.47629 (13)	0.0395 (3)	
O2	0.44050 (16)	0.34339 (9)	0.66510 (13)	0.0463 (3)	
O3	0.42447 (16)	0.38249 (9)	−0.03960 (14)	0.0448 (3)	
O4	0.24379 (16)	0.49243 (8)	−0.11360 (14)	0.0452 (3)	
O5	0.98177 (14)	0.19620 (8)	0.54419 (13)	0.0407 (3)	
H04	1.205 (3)	0.2741 (15)	0.711 (3)	0.057 (6)*	
H06	1.114 (3)	0.2797 (14)	0.857 (3)	0.054 (6)*	
H08	0.853 (3)	0.2351 (14)	0.857 (3)	0.053 (6)*	
H09	0.429 (3)	0.3798 (15)	−0.137 (3)	0.057 (6)*	

### Atomic displacement parameters (Å^2^)

**Table:**

	*U*^11^	*U*^22^	*U*^33^	*U*^12^	*U*^13^	*U*^23^
C1	0.0362 (8)	0.0364 (8)	0.0250 (7)	−0.0052 (6)	0.0080 (6)	−0.0025 (6)
C2	0.0300 (7)	0.0367 (8)	0.0280 (7)	−0.0046 (6)	0.0081 (6)	−0.0038 (6)
C3	0.0362 (8)	0.0488 (9)	0.0319 (8)	0.0001 (7)	0.0116 (6)	−0.0065 (7)
C4	0.0383 (9)	0.0506 (10)	0.0463 (10)	0.0095 (7)	0.0132 (7)	−0.0057 (8)
C5	0.0342 (8)	0.0458 (9)	0.0399 (9)	0.0051 (7)	0.0040 (7)	0.0013 (7)
C6	0.0292 (7)	0.0353 (8)	0.0306 (8)	−0.0035 (6)	0.0046 (6)	−0.0012 (6)
C7	0.0308 (7)	0.0361 (8)	0.0299 (8)	−0.0055 (6)	0.0016 (6)	0.0002 (6)
C8	0.0336 (8)	0.0387 (8)	0.0277 (7)	0.0037 (6)	0.0078 (6)	0.0043 (6)
N1	0.0303 (6)	0.0354 (7)	0.0256 (6)	−0.0017 (5)	0.0065 (5)	−0.0016 (5)
N2	0.0306 (7)	0.0398 (7)	0.0314 (7)	−0.0036 (5)	0.0087 (5)	0.0008 (5)
N3	0.0344 (7)	0.0675 (10)	0.0273 (7)	−0.0105 (7)	0.0095 (5)	−0.0106 (7)
N4	0.0356 (8)	0.0707 (11)	0.0383 (8)	−0.0115 (7)	0.0125 (6)	−0.0101 (7)
O1	0.0437 (7)	0.0467 (7)	0.0301 (6)	0.0099 (5)	0.0128 (5)	0.0059 (5)
O2	0.0561 (8)	0.0610 (8)	0.0245 (6)	0.0025 (6)	0.0153 (5)	0.0014 (5)
O3	0.0514 (7)	0.0597 (8)	0.0233 (6)	0.0161 (6)	0.0053 (5)	−0.0021 (5)
O4	0.0549 (7)	0.0471 (7)	0.0323 (6)	0.0058 (6)	0.0009 (5)	0.0057 (5)
O5	0.0393 (6)	0.0584 (7)	0.0262 (6)	−0.0001 (5)	0.0107 (4)	−0.0009 (5)

### Geometric parameters (Å, °)

**Table:**

C1—O2	1.2499 (18)	C7—O4	1.2107 (19)
C1—O1	1.2507 (19)	C7—O3	1.311 (2)
C1—C2	1.516 (2)	C8—O5	1.2338 (19)
C2—N1	1.3358 (19)	C8—N4	1.326 (2)
C2—C3	1.391 (2)	C8—N3	1.364 (2)
C3—C4	1.379 (2)	N2—N3	1.4090 (19)
C3—H3	0.9300	N2—H01A	0.8900
C4—C5	1.381 (2)	N2—H01B	0.8900
C4—H4	0.9300	N2—H01C	0.8900
C5—C6	1.387 (2)	N3—H08	0.88 (2)
C5—H5	0.9300	N4—H04	0.83 (2)
C6—N1	1.336 (2)	N4—H06	0.88 (2)
C6—C7	1.504 (2)	O3—H09	0.84 (2)
			
O2—C1—O1	124.90 (15)	O4—C7—C6	122.35 (15)
O2—C1—C2	117.83 (14)	O3—C7—C6	114.02 (13)
O1—C1—C2	117.25 (13)	O5—C8—N4	125.06 (15)
N1—C2—C3	122.73 (15)	O5—C8—N3	120.18 (15)
N1—C2—C1	116.04 (13)	N4—C8—N3	114.74 (14)
C3—C2—C1	121.22 (13)	C2—N1—C6	117.78 (13)
C4—C3—C2	118.65 (15)	N3—N2—H01A	109.5
C4—C3—H3	120.7	N3—N2—H01B	109.5
C2—C3—H3	120.7	H01A—N2—H01B	109.5
C3—C4—C5	119.29 (15)	N3—N2—H01C	109.5
C3—C4—H4	120.4	H01A—N2—H01C	109.5
C5—C4—H4	120.4	H01B—N2—H01C	109.5
C4—C5—C6	118.16 (15)	C8—N3—N2	116.86 (13)
C4—C5—H5	120.9	C8—N3—H08	121.6 (14)
C6—C5—H5	120.9	N2—N3—H08	116.0 (14)
N1—C6—C5	123.36 (14)	C8—N4—H04	117.1 (16)
N1—C6—C7	117.54 (13)	C8—N4—H06	116.7 (14)
C5—C6—C7	119.09 (14)	H04—N4—H06	126 (2)
O4—C7—O3	123.62 (15)	C7—O3—H09	108.9 (15)
			
O2—C1—C2—N1	−167.83 (14)	N1—C6—C7—O4	−176.25 (14)
O1—C1—C2—N1	10.7 (2)	C5—C6—C7—O4	4.7 (2)
O2—C1—C2—C3	11.3 (2)	N1—C6—C7—O3	4.6 (2)
O1—C1—C2—C3	−170.13 (15)	C5—C6—C7—O3	−174.44 (14)
N1—C2—C3—C4	−0.3 (2)	C3—C2—N1—C6	−1.2 (2)
C1—C2—C3—C4	−179.36 (15)	C1—C2—N1—C6	177.91 (13)
C2—C3—C4—C5	1.4 (3)	C5—C6—N1—C2	1.6 (2)
C3—C4—C5—C6	−1.1 (3)	C7—C6—N1—C2	−177.39 (13)
C4—C5—C6—N1	−0.5 (2)	O5—C8—N3—N2	−13.1 (2)
C4—C5—C6—C7	178.52 (15)	N4—C8—N3—N2	168.50 (15)

### Hydrogen-bond geometry (Å, °)

**Table:**

*D*—H···*A*	*D*—H	H···*A*	*D*···*A*	*D*—H···*A*
N2—H01A···O1^i^	0.89	2.00	2.7551 (17)	141
N2—H01A···N1^i^	0.89	2.21	2.9361 (17)	139
N2—H01B···O4^ii^	0.89	2.04	2.8781 (19)	156
N2—H01C···O1	0.89	1.84	2.7258 (18)	176
N4—H04···O2^iii^	0.83 (2)	2.22 (2)	2.993 (2)	155 (2)
N3—H08···O5^i^	0.88 (2)	2.06 (2)	2.8362 (19)	146.2 (19)
N3—H08···O1^i^	0.88 (2)	2.49 (2)	2.9319 (18)	112.0 (16)
O3—H09···O2^iv^	0.84 (2)	1.79 (2)	2.6109 (16)	165 (2)
N4—H06···O5^i^	0.88 (2)	2.05 (2)	2.869 (2)	153.5 (19)

Symmetry codes: (i) *x*, −*y*+1/2, *z*+1/2; (ii) −*x*+1, *y*−1/2, −*z*+1/2; (iii) *x*+1, *y*, *z*; (iv) *x*, *y*, *z*−1.
